# Urine Se concentration poorly predicts plasma Se concentration at sub-district scales in Zimbabwe, limiting its value as a biomarker of population Se status

**DOI:** 10.3389/fnut.2024.1288748

**Published:** 2024-02-07

**Authors:** Beaula Mutonhodza, Mavis P. Dembedza, Edward J. M. Joy, Muneta G. Manzeke-Kangara, Handrea Njovo, Tasiana K. Nyadzayo, R. Murray Lark, Alexander A. Kalimbira, Elizabeth H. Bailey, Martin R. Broadley, Tonderayi M. Matsungo, Prosper Chopera

**Affiliations:** ^1^Department of Nutrition, Dietetics and Food Sciences, University of Zimbabwe, Harare, Zimbabwe; ^2^London School for Hygiene & Tropical Medicine, London, United Kingdom; ^3^Rothamsted Research, West Common, Harpenden, United Kingdom; ^4^National Nutrition Unit, Ministry of Health and Child Care of Zimbabwe, Harare, Zimbabwe; ^5^School of Biosciences, University of Nottingham, Loughborough, United Kingdom; ^6^Department of Human Nutrition and Health, Lilongwe University of Agriculture and Natural Resources, Lilongwe, Malawi

**Keywords:** biomarkers, selenium deficiency, dietary selenium intake, estimated average requirement, iodothyronine deiodinase, micronutrient surveillance

## Abstract

**Introduction:**

The current study investigated the value of urine selenium (Se) concentration as a biomarker of population Se status in rural sub-Saharan Africa.

**Method:**

Urine and plasma Se concentrations were measured among children aged 6–59 months (*n* = 608) and women of reproductive age (WRA, *n* = 781) living in rural Zimbabwe (Murehwa, Shamva, and Mutasa districts) and participating in a pilot national micronutrient survey. Selenium concentrations were measured by inductively coupled plasma-mass spectrometry (ICP-MS), and urine concentrations were corrected for hydration status.

**Results:**

The median (Q1, Q3) urine Se concentrations were 8.4 μg/L (5.3, 13.5) and 10.5 μg/L (6.5, 15.2) in children and WRA, respectively. There was moderate evidence for a relationship between urine Se concentration and plasma Se concentration in children (*p* = 0.0236) and WRA (*p* = < 0.0001), but the relationship had poor predictive value. Using previously defined thresholds for optimal activity of iodothyronine deiodinase (IDI), there was an association between deficiency when indicated by plasma Se concentrations and urine Se concentrations among WRA, but not among children.

**Discussion:**

Urine Se concentration poorly predicted plasma Se concentration at sub-district scales in Zimbabwe, limiting its value as a biomarker of population Se status in this context. Further research is warranted at wider spatial scales to determine the value of urine Se as a biomarker when there is greater heterogeneity in Se exposure.

## 1 Introduction

Selenium (Se) is an essential human micronutrient and is a component of enzymes that regulate thyroid hormones responsible for healthy human development, growth, and cell metabolism (1). The iodothyronine deiodinase (IDI) enzyme assists in the production and function of triiodothyronine (T3) and thyroxine (T4) hormones (2). Selenium status is associated with the onset and progression of several diseases (including cancer, diabetes, Alzheimer’s disease, mental disorders, cardiovascular disorders, fertility impairments, inflammation, and infections) (3). Recent evidence suggests that high levels of selenium increase the risk of chronic diseases, such as diabetes, cancer, and neurodegenerative diseases (3, 4). Therefore, it is essential to maintain a safe range of selenium intake.

Various biomarkers and indicators of Se intake or status are used to assess the Se status of populations, including the concentration of Se in food, blood cells, hair, toenails, or body fluids (whole blood, plasma, or urine) and the concentration of selenoproteins or activity of selenoenzymes (5). The measurement of Se, either in hair or toenails, may provide an indication of whether the sampled population showed longer-term exposure to Se but is compromised due to the lack of accepted thresholds to define Se adequacy or deficiency and the high risk of contamination with exogenous Se from hair products or soil particles (6). Dietary assessment methods are challenging, mainly because of the large variability in the Se concentration of foods (7) relating to concentrations of plant-available Se in agricultural soils (8–10). The most useful and widely accepted biomarkers of Se intake/status are plasma, red blood cells, and whole blood Se concentration (8, 11). However, taking blood samples from large sample populations may incur high costs and is invasive, while in some contexts, there may be negative associations, myths, and misconceptions relating to blood sampling, including personal sensitivities regarding the use of blood samples and cultural beliefs (12–14), posing challenges to the use of blood Se concentration as a biomarker at the population level.

The measurement of urinary iodine concentration (UIC) in casual urine specimens is recommended for monitoring iodine status (15) and is routinely conducted in many settings. There may be opportunities to add Se analysis at relatively little cost to these existing surveys. Some studies have indicated that fasted urine samples, but not casual urine, may give a reasonable estimate of the urine output of Se on a population basis (16). However, other studies have validated single-void casual urine samples to be representative of population-level Se status (8, 12). In studies that have analyzed elemental concentrations of casual urine samples as a biomarker of status, challenges included variations in hydration state, fluid intake, physical activity, temperature, protein malnutrition, and genetic factors (17). For Se, specific gravity corrections can be applied to account for the effect of hydration status (urine dilution) on intra- and inter-individual variations in urine elemental concentrations (12, 18). Specific gravity correction works better than other Se correction factors, such as osmolality and creatinine (12), and has worked well for other nutritional surveillance applications using urine, including cadmium and steroid hormone concentrations (19, 20).

Dietary Se intake and plasma Se concentration correlate with daily urine Se excretion, especially in populations that live in Se-deficient areas (8, 21). Urine Se concentration reflects Se intake in a dose-dependent manner (22). Excess Se is excreted in urine, which might suggest that urine measurements are useful only in cases of high intakes and for identifying the risk of toxicity (23). However, there is increasing evidence that urine Se concentration reflects recent Se intake and may be a useful biomarker for assessing population-level Se status, even among Se-deficient populations (8, 12, 16, 21, 24). Current recommendations for assessing population urine elemental concentrations, for example, iodine using casual/spot urine samples developed by the World Health Organization (WHO), use the recommended daily urine output to extrapolate daily intakes (15, 25, 26). Globally, a systematic collation of the average 24-h urine volume is only available for children and adolescents aged 2–19 years (27). The typical urine output of pre-school-aged children is approximately 0.8 L/day (27) and that of healthy female adults is 1.5 L/day (28, 29), estimating that 50–60% of dietary Se is excreted in urine (30). In addition, Se intake determined using this estimate is suggested to be more accurate (16) or comparable to dietary assessment data (8, 27, 31, 32).

Urine Se excretion can be used to assess the population Se status by translating excretion into an estimate of dietary intake and then applying the estimated average requirement (EAR) cut-point method. The EAR is a daily nutrient level estimated to meet the requirements of half the healthy individuals in a particular life stage and gender group (28). The average intake (AI) for infants 7–12 months is 15 μg/day (7, 29, 33), the EAR for young children aged 1–6 years ranges from 17 to 23 μg/day (33, 34) and the EAR for non-pregnant women aged 15–50 years ranges from 45 to 50 μg/day (29, 31, 33, 34). Using an EAR cut-point approach, Joy et al. (35) quantified the Se deficiency risk due to the adequacy of Se in national food supplies across Africa to be 28%; greater risks of Se deficiency were estimated to occur in the Eastern (52%) and Central (49%) regions, followed by the Southern (26%), Northern (12%), and Western (6%) regions.

Including biomarkers of the micronutrient status in existing or planned national surveys or surveillance systems is a critical step in improving the capacity to promote, design, monitor, and evaluate micronutrient policies and programs (36). Estimates of micronutrient deficiencies (MNDs) using individual-level biomarker data are scarce, despite their value in determining population micronutrient status (37). Urine Se concentration data are scarce among Se-deficient populations. In Zimbabwe, food systems are localized, with a large proportion of diets coming from their own production or locally traded foods (38, 39), meaning that dietary exposures are closely related to the concentrations of plant-available Se in surrounding agricultural soils, and in these contexts, spatial variation in Se exposures at different scales is expected (9). The current study tested if casual urine is a practicable biomarker for measuring the Se status of children and WRA in a population where Se status is under strong geospatial control (40) by comparing casual urine Se status with plasma Se status. The study also examined the usefulness of urine Se concentration in predicting plasma Se concentration at different population scales: between district, enumeration areas (EAs) within district, and households (HHs) within EAs. The current study reports findings from a cross-sectional pilot micronutrient biomarker (blood and urine) survey, in which the aim was to inform national micronutrient surveillance methods. The study looked at the prevalence of Se deficiency in children aged 6–59 months and WRA from three predominantly rural districts: Murehwa (17.6502°S, 31.7787°E), Shamva (17.04409°S, 31.6739°E), and Mutasa (18.6155°S, 32.6730°E). These districts were chosen based on low dietary diversity, with 15–29.9% of children aged 6–59 months getting the minimum required food groups (41, 42), and to give variation in landscape.

## 2 Materials and methods

The current study assesses the usefulness of casual urine Se concentration as an alternative biomarker to plasma Se concentration, which has previously been reported (40), for the population-level Se status. The plasma Se study found that Se deficiency is widespread among women and children in all three rural districts. It was observed that children were more likely to be deficient than women, with 94.6% of children and 57.0% of women having plasma Se concentration below the IDI optimum activity threshold of 64.8 μg/L. The overall median (Q1, Q3) Se concentrations were 61.2 μg/L (48.7, 73.3) and 40.5 μg/L (31.3, 49.5) for children and women, respectively. Notable evidence of severe Se deficiency risk was observed in the sample population, with children recording plasma Se concentrations as low as 9.41 μg/L and 10.20 μg/L being the minimum in WRA (40). The urine and plasma samples were collected from individuals on the same day, allowing for comparison of urine and plasma at individual, household, EA, and district levels. Individuals within households and households within EAs are likely to have similar dietary patterns and environmental Se exposures, which may influence urine Se (12).

### 2.1 Sampling

Study design and methods are described in detail elsewhere (40, 43). The sampling design was nested at the level of the National Demographic Health Survey (DHS) sampling approach (44). Thirty enumeration areas (EAs) were selected per district. An EA contains an average of 120 households (44).

**Figure 1:**
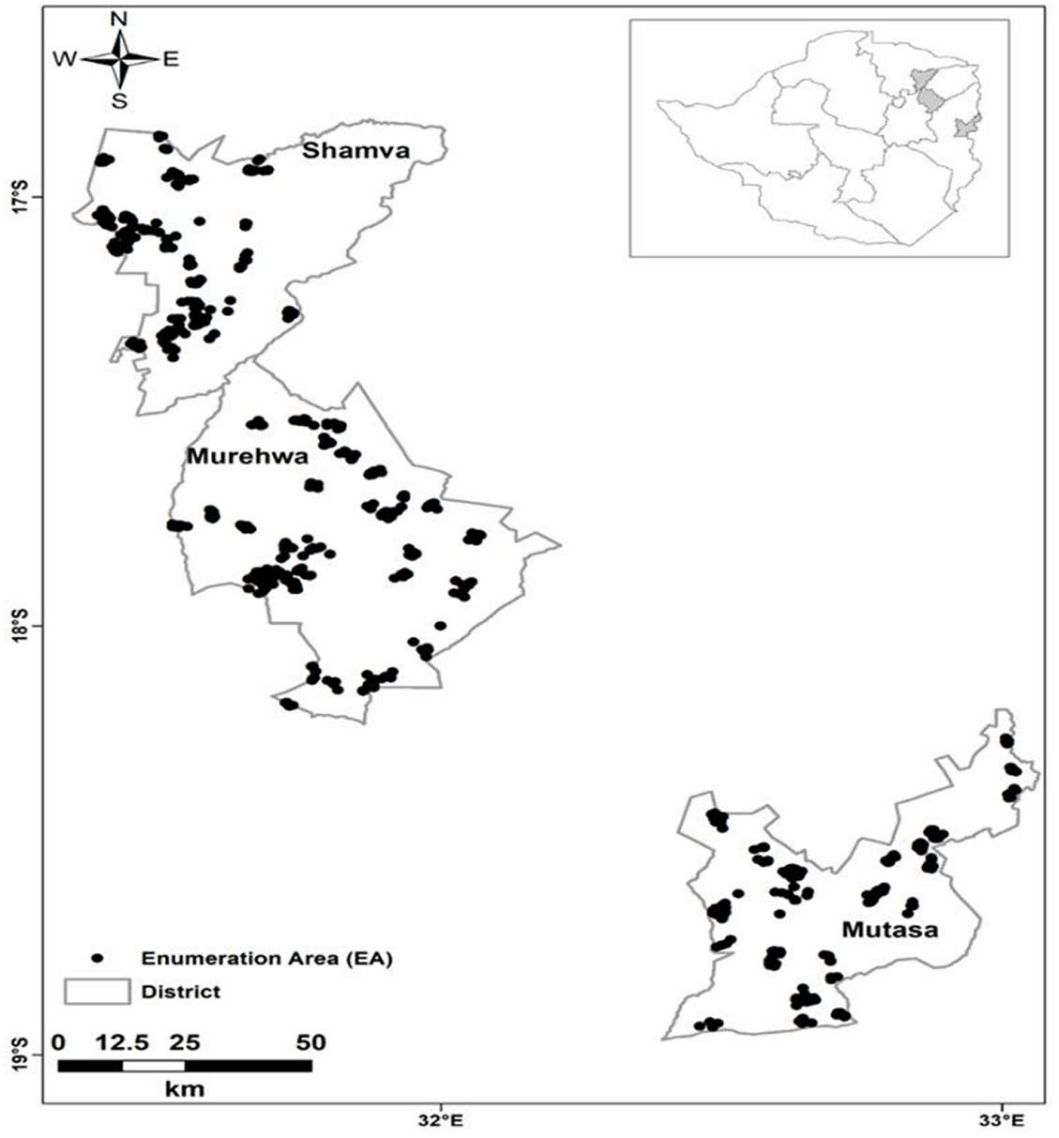
Sampling locations of eligible households (*n* = 900) from which study participants were recruited in Shamva, Murehwa, and Mutasa districts, rural Zimbabwe [adapted from Mutonhodza et al. (40)].

Probability proportional to size (PPS) based on the most recently recorded population survey (45) was used to select EAs. This was followed by eligible household listing, where households were eligible if there was a child aged 6–59 months and a woman aged 15–49 years. Households (*n* = 10) were selected by random systematic sampling without replacement in each EA. In households with multiple eligible child–WRA pairs, one pair was randomly selected using the Kish grid (46). The location of each household (sampling point) was determined using a global positioning system receiver (GPS) and verified through matched EA shape files (Figure 1). Recruited participants were directed to the nearest health facility for data and sample collection.

### 2.2 Urine sampling and analysis

One casual/spot urine sample (5 mL urine) was collected from children aged 6–59 months and WRA as per the World Health Organization (WHO) guidelines (11). A measure of specific gravity using a handheld temperature-corrected refractometer (PAL-10S, Atago, Tokyo, Japan) was determined in the field and confirmed in the laboratory before sample analysis. Urine was transported via a refrigerated cold chain to the University of Zimbabwe laboratory in Harare, where it was frozen at −80°C. Urine samples were transported on dry ice to the United Kingdom for elemental analysis using ICP-MS (Thermo-Fisher iCAP-Q, Thermo Fisher Scientific, Bremen, Germany). Samples were introduced at a flow rate of 1.2 mL/min from an autosampler (Cetac ASX-520) incorporating an ASXpress™ rapid uptake module through a perfluoroalkoxy (PFA) Microflow PFA-ST nebulizer (Thermo Fisher Scientific, Bremen, Germany). Sample processing was undertaken using Qtegra™ software (Thermo-Fisher Scientific), utilizing external cross-calibration between pulse-counting and analog detector modes when required. The instrument employs in-sample switching between two modes: (i) one mode using a collision cell charged with helium gas with kinetic energy discrimination (KED) to remove polyatomic interferences and (ii) another mode using hydrogen gas as the cell gas for Se analysis. Internal standards (scandium, germanium, rhodium, and iridium) were introduced on a separate line and used to correct for instrumental drift.

Calibration used a multielement (*n* = 30) solution in the concentration range of 0–100 μg/L (0, 20, 40, and 100 μg/L) (Claritas-PPT grade CLMS-2 from SPEX Certiprep Inc., Metuchen, NJ, USA). Accuracy was verified using appropriate certified reference materials (Seronorm™ L-1 (Lot 1,403,080, total Se certified value: 15.8 μg/L, recovery: 116%; 95% confidence interval: 12.6–19.0; *n* = 9) and Seronorm™ L-1 (Lot 1,706,877, total Se certified value: 10.5 μg/L, recovery: 113%; 95% confidence interval: 8.4–12.6; *n* = 1)). The limit of detection for Se was 0.065 μg/L based on a 3× standard deviation of 30 analytical blanks, and the limit of quantification was 0.216 μg/L based on a 10 × standard deviation of 30 analytical blanks. Urine Se levels for children and WRA were adjusted for hydration using the mean specific gravity of all participants (Equation 1), as follows:


(1) $$Se_{SG}=Se_{c}\;\left(SG_{\mathrm{Mean}}-1\right)/\left(SG_{Ind}-1\right)\text{,}$$

where $Se_{SG}$ is the specific gravity-corrected Se concentration, $Se_{c}$ is the uncorrected Se concentration, $SG_{\mathrm{Mean}}$ is the mean specific gravity of all (child or WRA) samples tested in the study, and $SG_{Ind}$ is the specific gravity measured for the individual samples.

### 2.3 Thresholds for urine Se concentration adequacy

Comparison of plasma Se concentration and urine Se concentration in the determination of Se deficiency prevalence at the population level was based on the lower limit of safe Se intake of 30 μg/day (30, 31), which corresponds to a baseline plasma Se concentration for the optimal activity of IDI (64.8 μg/L) (30, 47). Urinary Se excretion (μg/day) was derived from the conversion of urine Se concentration (μg/L) based on the recommended daily urine output (Equation 2). Generally, the mean/recommended urine output by the demographic group is 1 L/day in children under 5 years and 2 L/day in WRA (25, 31, 48). The total Se daily intake was estimated as twice the daily urine excretion (23, 31) (Eq. 3). The percentage of Se intake below the EAR approximated the proportion that was at risk of dietary inadequacy (34). An EAR cut-point of 20 μg/day was used for children aged 6–59 months based on average EAR for children aged 1–3 years (17 μg/day) and children aged 4–6 years (23 μg/day) (33, 34) and 50 μg/day for non-pregnant WRA (29, 31).


(2) $$Se_{\mathrm{cal}}=Uo\times Se_{SG}\text{,}$$

where $Se_{\mathrm{cal}}$ is calculated urine Se excretion (μg/day), Uo is the recommended daily urine output (children, 1 L/day; WRA, 2 L/day), and $Se_{SG}$ is the corrected urine Se concentration (μg/L).

Therefore, the estimated total daily Se intake (μg/day) is as follows:


(3) $$Se_{\text{int}}=2\times Se_{\mathrm{cal}}\text{,}$$

where $Se_{\text{int}}$ is the estimated total Se daily intake and $Se_{\mathrm{cal}}$ is the calculated urine Se excretion (μg/day).

### 2.4 Data analysis

Exploratory statistical analyses were conducted using SPSS version 20 (IBM, New York, United States) to examine the data and ensure there were no erroneous outlying values. A linear mixed model (LMM), which reflects the sampling design, was used to examine the relationship between plasma and urine Se concentration. This examination was done with the lme function from the nlme library for the R platform (49). After an initial exploratory fit of the model, the residuals were examined with summary statistics and plots to evaluate the plausibility of the assumption that they were normally distributed. It was found for the datasets of both the children and WRA that the assumption was plausible for a model with both plasma Se concentration and urine Se concentration (the dependent and independent variables, respectively) transformed to logarithms.

A “null” model with the only fixed effect, a constant mean value for (transformed) plasma Se concentration, was fitted in addition to the model with the mean linear function of (transformed) urine Se concentration (Supplementary material*: R-Script LMM*). This model allowed the effect of including the urine data as a predictor on the magnitude of the unexplained variation in plasma Se concentration to be examined at each level of the sampling design by comparing the corresponding variance components at the household, EA, and district levels of the sampling.

To compare the threshold for urine Se concentration with that for plasma Se concentration, we computed an indicator statistic that took the value 1 for cases where inferred urine Se excretion was less than 30 μg/day and a similar statistic that took the value 1 for cases where plasma Se concentration was smaller than the threshold of 64.8 μg/L. We then fitted a generalized linear mixed model using the glmer function in the lme4 library for the R platform (50), with the dependent variable as the indicator statistic for the deficiency by plasma Se and the fixed effect as a factorial variable with two levels corresponding to deficient and sufficient rates of urine Se excretion. In this model, the probability of deficiency by the plasma Se criterion is modeled from the fixed effects with the state of the plasma Se criterion treated as a binomial random variable. Adjustment factors (e.g., sex for children) were included in the generalized linear mixed model (Supplementary material: *R-Script GMM*).

### 2.5 Data and sample management

A temporary laboratory was established at each collection site to minimize contamination and facilitate accurate record keeping for the traceability of samples. Strict quality control measures were followed as guided by the Centre for Disease Control and Prevention (CDC) (51). All participants were assigned a unique numeric identity (ID) to label their samples; the labels were identifiable by district, demographic group, and destination. The IDs were also used on data capture forms, sample collection materials, and subsequent analyses to maintain participant anonymity. Specimen data were collected using passcode-protected tablets with KoboToolbox software (Android v2022.1.2). Data were reconciled and transferred daily to the central data processing server.

### 2.6 Ethical statements

The current study is a sub-component of a pilot micronutrient survey. The pilot survey was conducted in line with the Declaration of Helsinki. Ethical approval was obtained from the institutional review boards (IRBs) of the Medical Research Council of Zimbabwe (MRCZ/A/2664) and the Faculty of Medicine and Health Sciences, University of Nottingham (Reference#446-1912). Shipping permissions, including a material transfer agreement (MTA), were secured in Zimbabwe and the United Kingdom before sample shipping. Permission to conduct the research study in communities was obtained through consultative engagement with local government officials and the Ministry of Health at the provincial, district, clinic, and village levels. Written informed consent was obtained from all WRA, and consent was provided on the children’s behalf by the parent or legal guardian before data collection.

## 3 Results

**Figure 2:**
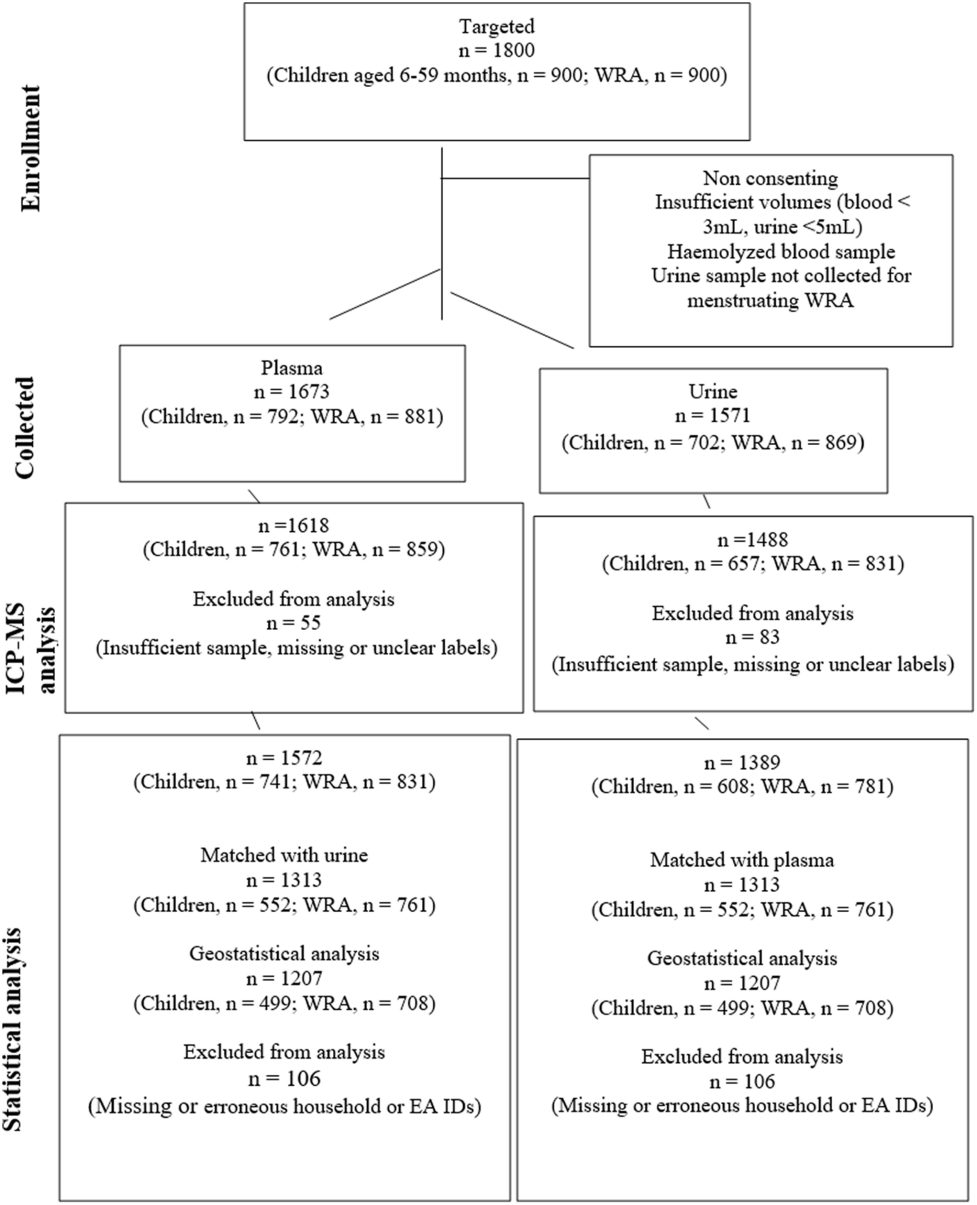
A CONSORT diagram showing the flow of participants, samples, and data from sampling to analysis. The reasons for sample collection failure were not quantified.

A total of 1,389 urine samples (children aged 6–59 months, *n* = 608; WRA, *n* = 781) were analyzed. In total, 1,313 plasma and urine samples (i.e., from the same individuals, children aged 6–59 months, *n* = 552; WRA, *n* = 761) were matched, and from these, samples with missing data, including erroneous household IDs, were excluded from the geostatistical analysis to account for changes in random effects at all scales, that is, between district, EAs within district, and HHs within EAs, yielding a total of 1,207 matched samples (499 child and 708 WRA) (see Figure 2).

### 3.1 Demographic characteristics

The sample size in the three districts was proportionate across the two demographic groups. The boy-to-girl ratio of the children was also proportionate (1:1) across districts. The median (Q1, Q3) age for the children was 29 months (18, 44) and that for women was 30 years (24, 37) (40). A few households (8.9%) earned a monthly household income adequate to meet the total consumption poverty line, set at USD$63.50 per person as of August 2021 (52). The main sources of household income were self-employment (26.3%), casual labor (19.5%), and cash crop farming (21.4%). Land size ownership was ≥5 ha for most of the households (61.2%). Maize (*Zea mays*) was the most predominant (60.2%) crop grown for consumption. A notably high prevalence of breastfeeding was observed in the sampled population, with 99.4 and 67.9% of children having been ever breastfed and exclusively breastfed, respectively. Vitamin A supplementation was high at >70%, while micronutrient powder supplementation coverage was low (9.1%) in children.

### 3.2 Urine Se status

A high prevalence of Se deficiency was indicated in children aged 6–59 months and WRA in all three rural districts (Table 1). Children were more likely to be deficient than WRA, with 96.9 and 74.4% of the population having urine Se excretion <30 μg/day, respectively. The concentration of Se in urine was lower in children compared to WRA with median (Q1, Q3) Se concentrations of 8.4 μg/L (5.3, 13.5) and 10.5 μg /L (6.5, 15.2), respectively. The overall median (Q1, Q3) calculated urine Se excretions were below the level suggested as adequate (30 μg/day) in both children and WRA, i.e., 8.4 μg/day (5.3, 13.5) and 21.1 μg/day (13.1, 30. 3), respectively. In children, the estimated median dietary intake (16.9 μg/day) was lower than the EAR (20 μg/day), as was that for WRA (42 μg/day) compared to the EAR (50 μg/day). The risk of inadequate dietary Se intake was lower in children (59.4%) compared to WRA (62.9%). Variations in urine Se concentrations were notable between districts. The estimated prevalence of Se deficiency was similar in Murehwa and Mutasa and greater in Shamva (Table 1).

**Table 1:** Urine Se status by district and demographic group (*n* = 1,389).

Group	District	*n*	Measured urine Se concentration (μg/L)^a^	Calculated urine Se excretion (μg/day)^b^	Estimated total Se daily intake (μg/day)^c^	Estimated prevalence (%) of Se deficiency	Estimated prevalence (%) of sub-optimal dietary Se intake
			Median (Q1, Q3)	Median (Q1, Q3)	Median (Q1, Q3)	Urine Se <30 (μg/day)	Estimated average requirement Children <20 (μg/day) WRA <50 (μg/day)
Children aged 6–59 months	Murehwa	237	7.6 (4.9, 12.0)	7.6 (4.9, 12.0)	15.2 (9.8, 24.1)	98.1	64.3
	Shamva	205	11 (6.4, 16.7)	11 (6.4, 16.7)	22 (12.8, 33.5)	94.9	47.7
	Mutasa	166	7.3 (5.0, 11.3)	7.3 (5.0, 11.3)	14.7 (9.9, 22.5)	97.9	66.9
	**Overall**	**608**	**8.4 (5.3, 13.5)**	**8.4 (5.3, 13.5)**	**16.9 (10.5, 27)**	**96.9**	**59.4**
Women of reproductive age (WRA)	Murehwa	276	9.8 (6.3, 13.9)	19.6 (12.5,27.9)	39.2 (25.1,55.8)	78.3	67.8
	Shamva	286	11.7 (7.1, 16.2)	23.4 (14.2, 32.3)	46.8 (28.5, 64.6)	69.9	58.0
	Mutasa	219	10.3 (6.2, 15)	20.6 (12.5, 30)	41.2 (25, 60)	75.3	63.0
	**Overall**	**781**	**10.5 (6.5, 15.2)**	**21.1 (13.1, 30.3)**	**42.1 (26.1, 60.7)**	**74.4**	**62.9**

a Measured urine Se concentration (μg/L) was corrected for hydration status using the combined mean specific gravity of both groups (1.0254 kg/m^3^).

b Calculated urine Se excretion (μg/day) was based on the recommended urine output (children aged 6–59 months, 1 L/day; WRA, 2 L/day).

c Estimated total Se daily intake (μg/day) was based on urine excretion of 50% of Se intake.

### 3.3 Selenium deficiency indicated by urine and plasma Se concentrations

The generalized linear mixed model for the probability of Se deficiency indicated by plasma Se concentration did not show a significant effect for the urine Se concentration factor (above or below the 30 μg/day excretion rate threshold) in the case of children (*p* = 0.88). There was evidence for a significant effect in the case of the data for WRA (*p* < 0.0001). However, the unexplained variation in the model remains large, and the fitted probability that an individual appears deficient by the plasma threshold if they exceed the threshold urine concentration was 0.38, whereas the fitted probability if they are below the urine threshold was 0.60. This fact shows that the model, while statistically distinguishable from random prediction, would have a large error rate.

### 3.4 Urine Se as a predictor of plasma Se

**Figure 3:**
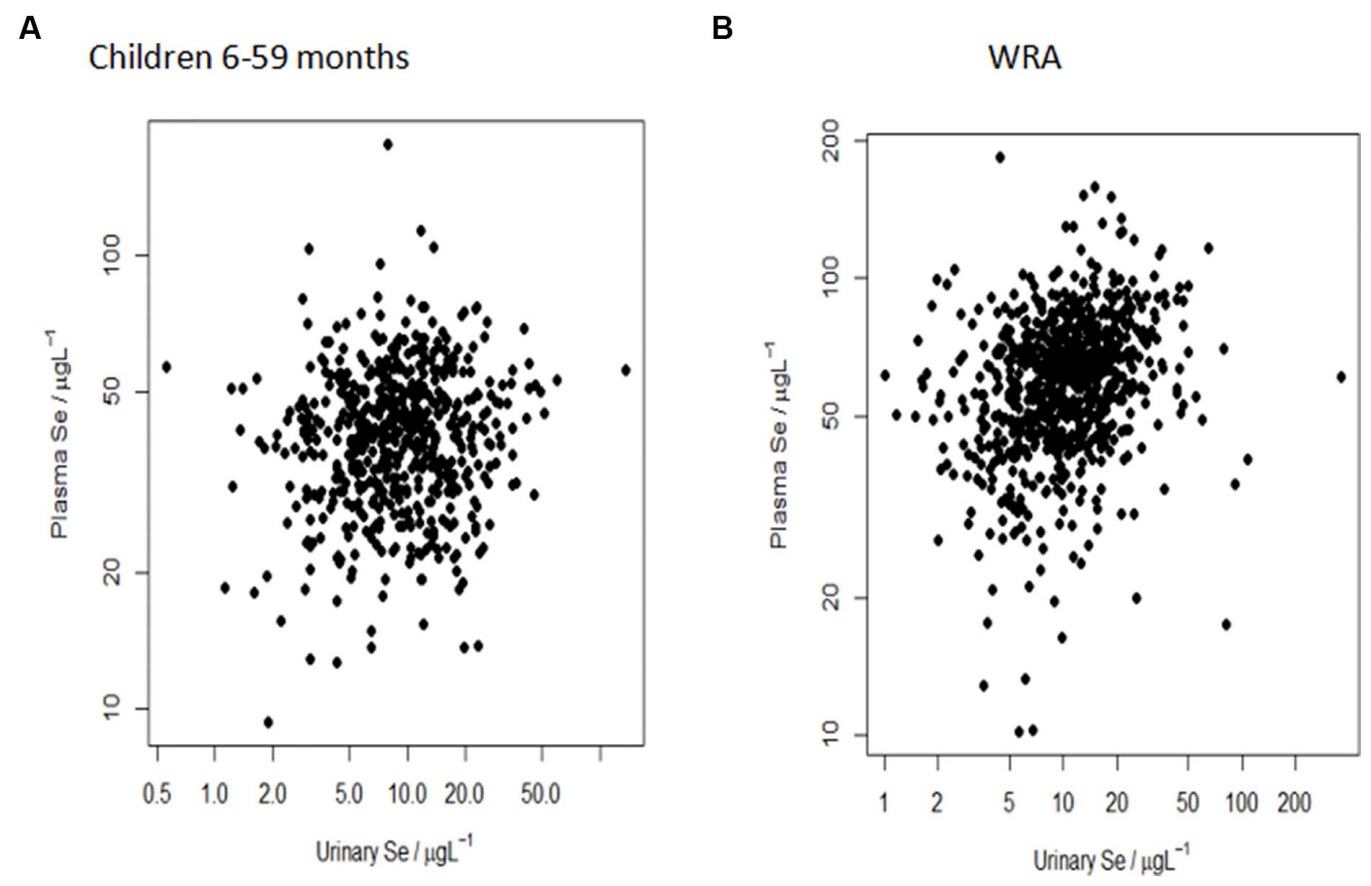
Null correlation between plasma Se concentration (μg/L) and urine Se concentration (μg/L) in **(A)** children aged 6–59 months and **(B)** WRA at the sub-district population level. The more spread out the points, the weaker the relationship.

There was a significant positive relationship between urine Se concentration and plasma Se concentration in both children aged 6–59 months (*p* = 0.0236) and WRA (*p* = < 0.0001). However, the reduction in the variance at any scale on adding urine Se as a predictor was small for both WRA and children (Table 2). The increases in the variance component in some cases may reflect an estimation error of a small effect and some shifting of the error between scales. Overall, the correlation between plasma Se concentration and urine Se concentration was null, as indicated by the widespread scatter plot pattern (Figure 3), with no evidence of a marked reduction of the variance component on adding urine Se as a predictor for a relationship at any level of the sampling design (Table 2).

**Table 2:** Urine Se as a predictor of plasma Se at the population level by a linear mixed model.

Demographic group	Level	Variance component, plasma Se	
		Null model^a^	Urine Se^b^
Children aged 6–59 months	Between district	0.014	0.018
	EAs within district	0.011	0.008
	HHs within EAs	0.092	0.097
	Residual	0.014	0.014
Women of reproductive age (WRA)	Between district	0.022	0.024
	EAs within district	0.013	0.011
	HHs within EAs	0.079	0.075
	Residual	0.012	0.011

a Model just examines the variance components for plasma Se.

b Model has urine Se as a predictor.

## 4 Discussion

### 4.1 Context of the study

Selenium status has never been analyzed in national micronutrient or demographic and health surveys in Zimbabwe. However, casual urine samples are routinely collected for iodine assessments as part of the national MND surveillance system. Multi-mineral analysis of urine samples, including Se, may provide an opportunity for expanding population-level micronutrient surveillance without additional participant burden and at a small additional analytical cost compared to the cost of running an additional survey. Zimbabwe has programs such as the micronutrient powder supplementation program that have never been evaluated using biomarkers since their inception in 2017, and this lack of evaluation represents a research gap. This study was designed to evaluate the use of casual urine as an indicator for assessing the Se status of children aged 6–59 months and WRA in rural Zimbabwe.

### 4.2 Overview of the current study and summary of the findings

Therefore, our study compared the prevalence of Se deficiency based on urine Se concentrations to the prevalence estimated from plasma Se concentrations. The results will be useful to validate the use of urine Se concentration in the prediction of Se deficiency at the population level.

There was no evidence that the urine Se status of an individual child (relative to a threshold implying an inadequate rate of Se excretion) is predictive of their Se status, as indicated by the plasma concentration. There was evidence of such a relationship in the case of WRA, but the predictive error of the model was large. The concentration of Se in casual urine samples was statistically related to its concentration in plasma in children aged 6–59 months (*p* = 0.0236) and WRA (*p* = <0.0001) in rural Zimbabwe, but the relationship had poor predictive value. These results show that urine and plasma Se concentrations are related but do not provide evidence that the urine Se concentration is predictive of plasma Se status if this is regarded as a “gold standard” biomarker for Se status.

### 4.3 Selenium deficiency urine vs. plasma

Our findings show that all three districts have large risks of Se deficiency and suggest a high risk of severe Se deficiency that may be detrimental to health among most children and approximately three-quarters of WRA. Our results indicate that casual urine predicts Se deficiency better in WRA compared to children aged 6–59 months based on the probability of deficiency from whether the urine excretion rate is above or below the “deficient” threshold of 30 μg/day (consistent with the LMM results). Comparably, in Malawi, urine Se concentration was a useful biomarker for assessing the population-level Se status of some groups, particularly WRA (12).

The observed median values of urine Se concentration for children (8.4 μg/L) were lower in the current study than that reported in the study of Malawi (12). The median values of urine Se concentration in WRA (10.5 μg/L) were also lower compared to those reported in other countries, namely New Zealand (32 μg/L) (31) and Malawi (16.9 μg/L) (12), suggesting that the risk of Se deficiency in children aged 6–59 months and WRA in the three rural Zimbabwean districts is greater than that in Malawi and corresponds with the plasma Se results of the same studies (13, 40). Our findings suggest that hydration-corrected Se concentrations in casual urine samples can indicate low Se exposures and, therefore, a high risk of deficiency, which, thus, may be useful for the surveillance of Se status in national MNDs monitoring and surveillance systems, where urine samples are already collected for iodine assessment. In Zimbabwe, urinary iodine is used to track coverage of iodized salt (53), and the population distribution of UIC gives a good indication of the population’s iodine status. Similarly, Se concentration in casual urine samples may be valuable to track the effectiveness of an intervention to increase Se status, but only where this leads to a marked change in exposure, for example, a supplementation or agronomic biofortification program with good reach and adherence.

### 4.4 Urine Se concentration as a predictor of plasma Se concentration at the population scale

A significant relationship was observed between urine Se concentration and plasma Se concentration. However, the value for prediction is not just a matter of the presence of an effect but also about the practical value of reducing uncertainty. The variance components in the current study show that any such effect is negligible. Based on these findings, we can conclude that urine Se concentration is not a good predictor of plasma Se concentration at any population scale measured within this pilot survey: between district, EAs within district, and HHs within EAs. These findings validate plasma as the primary biomarker of Se status at the population level. Thus, we can conclude that plasma remains the principal biomarker, but urine might be used as an alternative biomarker to reflect a snapshot of the general distribution and magnitude of population Se status when data from plasma samples are unavailable and resources to generate these data are limited.

### 4.5 Dietary Se intake as estimated by urine Se concentration

The current findings indicate that the estimated median excretion of Se was lower than the level suggested as adequate (30 μg/day) for both children (8.4 μg/day) and WRA (21.1 μg/day). Overall, a proportionate percentage of children (59.4%) and WRA (62.9%), respectively, had estimated Se intakes below the recommended EAR (children aged 6–59 months, 20 μg/day; WRA, 50 μg/day). Selenium intake inadequacies have also been reported in other African countries. Among 46 countries across continental Africa, Joy et al. reported mean and median supplies of Se in national food systems of 50 and 55 μg/capita/day (35). Selenium in national food supplies tends to be lower in Southern and Central Africa (8). Intakes of 17 μg/day were reported for adults in rural Burundi (54) and 15–21 μg/day for children in the rural areas of Zomba District, Malawi (55). These low Se intakes correspond with the widespread Se deficiency observed in rural children and WRA in the current study, as well as in Malawi (13). Both countries (Zimbabwe and Malawi) have high maize consumption, making the crop a major contributor to dietary Se intake (56, 57). The average maize consumption for adults in Zimbabwe, estimated from national food supplies, is over 250 g/person/day (58, 59), and a high reliance on starchy staples increases the risk of dietary MNDs, including Se (59). Previously, sub-optimal maize Se concentrations were observed in Malawi (56), South Africa (60), and Zambia (61). Cognizant of the similarities in geography and diet between Zimbabwe and these African countries (62) and also given that Zimbabwean soils are predominantly acidic (63–65), there is a high likelihood that the Zimbabwean maize crop has low Se concentrations (56), which could result in Se deficiency in the human population (66). Direct crop and dietary Se assessments are warranted to validate these findings.

## 5 Limitations of study

Our survey population may have had low heterogeneity of Se exposure relative to a national survey since it was a pilot covering only three predominantly rural districts. Phiri et al. (12) showed that urine Se concentration may be useful for providing sub-national insights into variation in Se status when there is sufficient heterogeneity of exposures. Additionally, no urine Se concentration (μg/L) cutoff points are published to assess the magnitude or severity of Se deficiency at the population level. The current study only measured urine Se concentration and derived Se intakes by extrapolation. Although correlations exist between dietary Se intake and urine Se concentration, extrapolation may overestimate intakes due to the variation between the recommended and the typical urine output when casual urine is measured. Nevertheless, the present study contributes to the limited body of knowledge on urine Se concentration among Se-deficient populations, indicating that casual urine samples poorly predict plasma Se concentration at sub-district scales and validate the need for a national-level micronutrient biomarker survey in Zimbabwe.

## 6 Conclusion

Selenium concentrations in paired urine and plasma samples were correlated among children aged 6–59 months and adult women in rural Zimbabwe, although the relationships were weak, particularly among children. The application of urine Se concentration as a biomarker is unlikely to be useful to predict individual Se status or to distinguish variation in population Se status at the enumeration area level. Plasma Se concentration, therefore, remains the preferred biomarker of population-level Se status. However, urine Se concentration may be a useful biomarker at district-national level scales, particularly in populations with highly heterogeneous exposure as observed in Malawi, and further research is needed to determine its value at the national level in Zimbabwe.

## Data availability statement

The original contributions presented in the study are included in the article/Supplementary material, further inquiries can be directed to the corresponding authors.

## Ethics statement

The studies involving humans were approved by Institutional Review Boards (IRBs) of the Medical Research Council of Zimbabwe (MRCZ/A/2664) and the Faculty of Medicine and Health Sciences, University of Nottingham (Reference#446-1912). The studies were conducted in accordance with the local legislation and institutional requirements. Written informed consent for participation in this study was provided by the participants’ legal guardians/next of kin.

## Author contributions

BM: Conceptualization, Data curation, Formal analysis, Investigation, Methodology, Project administration, Writing – original draft. MD: Project administration, Writing – review & editing. EJ: Validation, Visualization, Writing – review & editing. MM-K: Writing – review & editing, Project administration. HN: Writing – review & editing, Resources. TN: Writing – review & editing, Project administration, Resources. RL: Formal analysis, Validation, Writing – review & editing. AK: Writing – review & editing, Methodology, Supervision. EB: Writing – review & editing, Data curation, Methodology, Supervision, Validation. MB: Writing – review & editing, Funding acquisition, Resources, Validation. TM: Validation, Visualization, Writing – review & editing, Investigation, Methodology, Project administration, Supervision, Conceptualization, Data curation. PC: Project administration, Writing – review & editing, Funding acquisition, Investigation, Methodology, Resources, Supervision, Validation, Visualization, Conceptualization, Data curation.
